# Comparison of Functional Outcome and Stroke Recurrence in Patients with Embolic Stroke of Undetermined Source (ESUS) vs. Cardioembolic Stroke Patients

**DOI:** 10.1371/journal.pone.0166091

**Published:** 2016-11-10

**Authors:** Antonio Arauz, Eugenia Morelos, Jonathan Colín, Javier Roldán, Miguel A. Barboza

**Affiliations:** 1 Stroke Clinic, Instituto Nacional de Neurología y Neurocirugía Manuel Velasco Suárez, Mexico City, Mexico; 2 Instituto Nacional de Cardiología Ignacio Chávez, Mexico City, Mexico; Henry Ford Health System, UNITED STATES

## Abstract

**Background:**

Embolic stroke of undetermined source (ESUS) recurrence and functional outcome from long-term follow-up is not well delineated. The purpose of this study is to compare these functional variables between ESUS vs. cardioembolic stroke (CS) patients.

**Methods:**

We analyzed data of consecutive ESUS and CS patients from our institutional database, from January 2003 until April 2015. The endpoints were stroke recurrence, mortality and poor clinical outcome (Modified Rankin Score 3–6), at discharge, 6 months and final follow-up. Adjusted multivariate Cox analysis and Kaplan-Meier curves were used to estimate the probability of recurrence and death.

**Results:**

149 ESUS (median age 44 years) and 235 CS (median age 66 years) consecutive patients were included in the study. Median follow-up period for the entire sample was 19 months (interquartile range 6.0–45.0 months). Stroke recurrence was similar between ESUS and CS patients (5.4% vs. 9.8% respectively, p = 0.12). Death occurred in 30 CS cases (12.8%), with a cumulative probability of survival of 77%. Poor functional outcome was present in 58.3%, 54.0% and 54.9% at discharge, 6 months and final follow-up respectively in CS patients, significantly worst compared to ESUS cases (HR 3.1; CI 95% 1.96–4.68). Oral anticoagulation presents with a HR 8.01 for recurrence, and antiplatelet therapy had the highest risk for recurrence for both groups (HR 24.3).

**Conclusion:**

ESUS patients are substantially younger than CS patients but have a stroke recurrence rate similar to CS patients, with a lower mortality rate, and better functional outcome on long-term follow-up.

## Introduction

Considering TOAST classification for ischemic stroke subtypes,[1] strokes of undetermined origin (25% of all ischemic strokes with complete study protocols),[2] could be related to a thromboembolic origin mainly caused by paroxysmal atrial fibrillation and much less by other potential sources of embolism.[3] This population has received special attention, due to the lack of evidence in specific preventive or therapeutic approaches, despite their incidence of recurrence, which has been reported nearly 1–2% per year in young stroke population,[3–6] and as high as 7.8% in older patients.[3,7–9] Data from certain trials, have reported that this rate of recurrence could be related to underlying atrial fibrillation or other cardiac sources of embolism, which leads to the rationale that long term cardiac monitoring strategies could be helpful to detect them.[10,11]

A recent clinical construct known as embolic stroke of undetermined source (ESUS) has been introduced by the Cryptogenic Stroke/ESUS International Working Group, with the goal of identify patients who have clinical and radiological characteristics of embolism, but despite a minimum diagnostic workup the source of this embolism could not be confirmed.[2] In a substantial fraction of ESUS patients, cardiogenic embolism is likely to be the causes. ESUS patients are, on average, relatively young with non-disabling strokes.[12] Little information is currently available regarding stroke recurrence or functional condition in long-term follow-up studies for these patients, with only a single study reporting a cumulative probability of stroke recurrence of 29%, for a mean follow-up of 30.5±24.1 months.[13]

The aim of the present study is to compare functional outcome and stroke recurrence from an ESUS population compared with recognized cardioembolic stroke (CS) patients, derived from a large prospective Mexican stroke registry.

## Materials and Methods

The Instituto Nacional de Neurología y Neurocirugía (INNN) Stroke Registry includes consecutive patients with an acute first-ever confirmed ischemic stroke admitted to this Institute in Mexico City. The study population was obtained from patients admitted between January 2003 and April 2015.

This registry includes detailed data prospectively recorded, including demographics, medical history and associated cardiovascular risk factors, current medication, time of stroke onset and hospital admission, in-hospital stay, stroke characteristics, functional outcome (established by the modified Rankin score [mRs]), imaging and laboratory tests and treatment. Stroke severity was assessed according to the National Institute Health Stroke Scale score (NIHSS) at admission and hospital discharge.

From our registry, ESUS patients were retrospectively selected, according to the criteria proposed by the Cryptogenic Stroke/ESUS International Working Group,^2^ which defines ESUS as a stroke detected by CT or MRI that is not lacunar, absence of extra cranial or intracranial atherosclerosis causing ≥50% luminal stenosis in arteries supplying the area of ischemia (detected by CT angiography or cervical ultrasonography), no major-risk cardioembolic source of embolism (discarded by transthoracic echocardiogram and at least 24-hours cardiac holter) and no other specific cause of stroke identified. Lacunar stroke was defined as a tomographic subcortical infarct smaller than or equal to 1.5 cm (≤2.0 cm on MRI diffusion images) in largest dimension, in the distribution of the small, penetrating cerebral arteries.[1,2]

Cardioembolic stroke was established with the presence of major-risk cardioembolic sources for stroke (according to ASCOD phenotyping classification system, C1), defined as: permanent or paroxysmal atrial fibrillation, sustained atrial flutter, intracardiac thrombus, prosthetic cardiac valve, atrial myxoma or other cardiac tumours, mitral stenosis, recent (<1 month) myocardial infarction, left ventricular ejection fraction less than 30%, valvular vegetations, or infective endocarditis. Patent foramen ovale was excluded if no thrombus in situ, concomitant pulmonary embolism or proximal deep venous thrombosis preceding the index cerebral infarction, were detected to be classified as C1.[14]

Hypertension was defined as systolic blood pressure >140 mm Hg or diastolic blood pressure >90 mmHg diagnosed at least twice before stroke or if patient was already under anti-hypertensive drugs. Diabetes mellitus was detected if the patient was already on anti-diabetic drugs/insulin or if fasting blood glucose level was >126 mg/dL before stroke. Dyslipidemia was defined as total cholesterol concentration >200 md/dL the day after admission or if patient had a previous diagnosis of dyslipidemia. Coronary heart disease was established if the patient had previous diagnosis of myocardial infarction, or if it was present preceding the index cerebral infarction. Transient ischemic attack was defined as complete disappearance of neurologic signs and symptoms within 24 hours, without signs in neuroimaging. Stroke was defined according to the World Health Organization criteria. Patients with recurrent stroke previous to the study period were not included in the protocol.

The comparative group included stroke patients classified as cardioembolic stroke (C1) according to ASCOD classification system, in the same period of study from the INNN registry; therefore all of them underwent the same standardized diagnostic and treatment follow-up.

Primary outcome of the study was ischemic stroke recurrence, which was defined as a new cerebrovascular event, with new neurological deficit or increasing in the previous ones, lasting more than 24 hours, and subsequent confirmation with brain imaging studies, that confirms the presence in a different vascular territory. Secondary outcomes were death (due to the index or recurrent stroke, myocardial infarction or systemic embolism) and poor functional outcome (defined as mRs 3–6). Follow-up period included clinical assessments (from vascular neurologists at the Stroke Clinic) at discharge, 6 months at discharge and final evaluation at the end of the study period. Death was confirmed at discharge death certificates or by telephone interview to relatives from those patients who died during the follow-up period.

The local Ethics Committee approved the scientific use of the data collected in this Stroke Registry, and no informed consent was necessary due to the nature of the stroke registry.

### Statistical analysis

All statistical analyses were done with IBM SPSS Statistics for Macintosh, version 22.0 (IBM Corp, Armonk, NY, USA). Categorical variables are expressed as percentages. Continuous variables are expressed as means with standard deviation (SD) or medians with interquartile range (IQR), according to normality tests (Kolmogorov-Smirnov and Shapiro-Wilk). Categorical variables were compared with chi-square for both populations (ESUS vs. CS), and for continuous variables comparison, T-Student or U Mann-Whitney tests were used according to normality distribution. Cox proportional hazard models (univariate and multivariate) were constructed to analyze poor outcome, death and recurrence in the study period, adjusted for variables considered as confounders for outcome (sex, comorbidities, therapeutic approach after the index stroke [oral anticoagulation or antiplatelet therapy] and age); hazard ratios (HR) and 95% confidence intervals (95% CI) are provided. For data lost during the follow-up period, this were censored at the last time known to be alive, if no telephone interview was possible. Kaplan-Meier curves were plotted for outcomes on each group, compared with long rank test. Statistical comparisons or interactions with p<0.05 were considered statistically significant.

## Results

INNN Stroke Database for the study period (January 2003—April 2015) included a total amount of 1673 ischemic stroke patients; from this sample, a total of 308 were classified primarily as cardioembolic strokes and 216 patients were confirmed for cryptogenic strokes.

From the cryptogenic sample, 149 (68.9%) patients fulfilled the inclusion criteria for ESUS, meanwhile 67 were ruled out due to: incomplete follow-up information (60 cases) and medical files not available (7 cases). The ESUS population corresponds to 8.9% from the entire stroke cohort.

From the CS sample (308 patients), only 235 patients fulfilled the inclusion criteria, meanwhile 73 were ruled out due to: incomplete follow-up information (12 cases), incomplete medical/laboratory information on medical files (12 patients) or cases with previous diagnosis of cardioembolism with conditions classified as ASCOD C2 or C3,[14] (49 cases). CS cases included atrial fibrillation (136 cases), dilated cardiomyopathies with left ventricle ejection fraction <35% (21 cases), mechanical valve (17 cases), mural thrombus in left atrium (13 cases) or left ventricle (4 cases), myocardial infarction within 4 weeks preceding the index stroke (10 cases), confirmed paradoxical embolism (9 cases) myxoma (4 cases) and other C1 causes 22 cases.

Follow-up period for the entire sample was 19 months (interquartile range [IQR] 6–45 months), with a median time for ESUS of 28 months (IQR 10.5–49.0 months), and 25 months (IQR 3.0–39.0 months) for CS cases (p<0.001). The study included 194 female patients (50.5%). The median age for the entire population was 57 years (IQR 44–77 years-old). ESUS patients were younger (median age 44 years) than CS (median age 66 years), p<0.001. Main demographic data can be seen on Table 1.

**Table 1:** Demographic characteristics from ESUS and CS patients.

	Total N = 384 (%)	ESUS n = 149 (%)	CS n = 235 (%)	P value
Age, years (median, IQR)	57 (44.0–70.0)	44 (30.5–57.0)	66 (54.0–75.0)	<0.001*
Follow-up,months (median, IQR)	19 (6.0–45.0)	28 (10.5–49.0)	25 (3.0–39.0)	<0.001*
Female	194 (50.5)	73 (49.0)	121 (51.5)	0.63
**Main risk factors**				
Hypertension	166 (43.2)	37 (24.8)	129 (54.9)	<0.001
Diabetes	62 (16.1)	14 (9.4)	48 (20.4)	0.004
Hypercholesteromia	36 (9.4)	14 (9.4)	22 (9.4)	0.99
Smoking	90 (23.4)	44 (29.5)	46 (19.6)	0.02
Ischemic cardiopathy	40 (10.4)	1 (0.7)	39 (16.6)	<0.001
Alcohol intake	66 (17.2)	29 (19.5)	37 (15.8)	0.35
Previous TIA	7 (1.8)	0 (0)	7 (3.0)	0.03
**Treatment**				
IV thrombolysis	28 (7.3)	8 (5.4)	20 (8.5)	0.25
Oral anticoagulation	159 (41.4)	8 (5.4)	151 (64.3)	<0.001
Antiplatelet	205 (53.4)	135 (90.6)	70 (29.8)	<0.001
**Functional outcome**				
Initial NIHSS (median, IQR)	8 (4–14)	7 (3–10)	9 (5–16)	0.001*
mRs (3–6) discharge	196 (51.0)	59 (39.6)	137 (58.3)	<0.001
mRs (3–6) 6 months	176 (45.8)	49 (32.9)	127 (54.0)	<0.001
mRs (3–6) final follow-up	170 (44.3)	41 (27.5)	129 (54.9)	<0.001
Death	30 (7.8)	0 (0)	30 (12.8)	<0.001
Recurrence	31 (8.1)	8 (5.4)	23 (9.8)	0.12
Time to recurrence in months (median, IQR)	11 (1.5–24.0)	3 (1.0–20.0)	14 (3.5–24.0)	0.16*

*U Mann-Whitney Test. ESUS: embolic stroke of undetermined source; CS: cardioembolic stroke; TIA: transient ischemic attack; IQR: interquartile range; mRs: modified Rankin score

Regarding risk factors in CS patients, hypertension (54.9%), diabetes (20.4%), ischemic cardiopathy (16.6%) and previous transient ischemic attack (3%), were statistically significant compared with ESUS cases, meanwhile smoking was slightly more prevalent in ESUS patients (29.5%). From the CS patients 64.3% were treated with oral anticoagulation, 29.8% with antiplatelet drugs, meanwhile the majority of ESUS patients received antiplatelet drugs (90.6%), and 8 (5.4%) patients received oral anticoagulation. Intravenous thrombolysis was administered in 7.3% cases of the entire stroke sample.

### Functional outcome

NIHSS from CS cases were higher (median 9.5, IQR 5–16) compared to ESUS cases (p = 0.001). Poor functional outcome (mRs 3–6) was present in 58.3%, 54.0% and 54.9% at discharge, 6 months follow-up and final follow-up respectively in CS patients, which was statistically significant compared with ESUS cases. 30 deaths were recorded during the study period, all of them from the CS arm, with cumulative probability of survival of 77%. Recurrence was present in 23 (9.8%) CS cases, vs. 8 (5.4%) in ESUS patients, with statistical difference among groups. Time to recurrence was higher in CS patients (median 14 months, IQR 3.5–24), with a cumulative prevalence of stroke recurrence of 10% for the ESUS population vs. 18% in the CS population (p = 0.12).

Recurrent cases were also studied to provide further analysis from the ethiology of the recurrence. Cardioembolic strokes remained as cardioembolic as the causative condition (16 cases were under oral anticoagulation, and 7 cases did not received anticoagulation). From those ESUS recurrent cases, 6 cases remained as cryptogenic after subsequent further studies for ethiology, and 2 of them were classified as possible intracranial atherosclerotic. Only 4 (12.9%) recurrent cases died during the study; there was no significant difference for mortality during recurrence between cardioembolic and ESUS cases (OR 1.8, CI 95% 0.6–5.7; P = 0.27).

**Fig 1:**
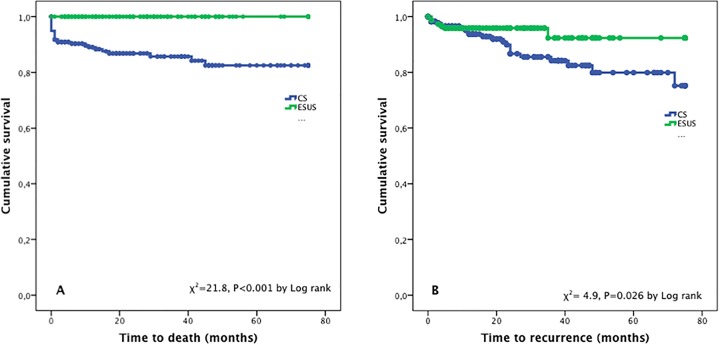
Kaplan-Meier curves for (A) mortality and (B) recurrence in ESUS vs CS patients.

Kaplan-Meier curves for the risk of death (χ^2^ = 21.8, p<0.001 by log rank), and recurrence (χ^2^ = 4.9, p<0.026 by log rank), are provided in Fig 1.

Multivariate adjusted Cox models for poor outcome (mRs 3–6) and recurrence are summarized in Table 2. CS cases (HR 3.1; CI 95% 1.96–4.68), oral anticoagulation (HR 4.63; CI 95% 2.42–8.86) and antiplatelet therapy (HR 3.9; CI 95% 2.04–7.52) after adjustment had the highest association for poor outcome (mRs 3–6); meanwhile antiplatelet therapy (HR 24.3, CI 95% 1.82–324.6) had the highest association for recurrence.

**Table 2:** Adjusted Cox regression models for final follow-up bad outcome (mRs 3–6) and recurrence in ESUS vs CS patients (final model).

	mRs 3–6		Recurrence	
	HR (CI 95%)	P value	HR (CI 95%)	P value
Cardioembolic	3.10 (1.96–4.68)	<0.001	0.39 (0.11–1.34)	0.13
Female	0.78 (0.56–1.09)	0.15	1.01 (0.43–2.33)	0.99
Hypertension	0.76 (0.55–1.06)	0.10	0.71 (0.27–1.90)	0.50
Diabetes	1.12 (0.77–1.82)	0.42	0.94 (0.27–3.27)	0.92
Smoking	1.23 (0.81–1.89)	0.33	1.47 (0.48–4.45)	0.49
Ischemic cardiopathy	0.56 (0.35–0.89)	0.01	2.60 (0.22–30.1)	0.44
Thrombolysis	1.04 (0.54–1.99)	0.91	1.20 (0.30–4.74)	0.79
Oral anticoagulation	4.63 (2.42–8.86)	<0.001	8.01 (0.68-94-3)	0.09
Antiplatelet	3.9 (2.04–7.52)	<0.001	24.3 (1.82–324.6)	0.01

## Discussion

Long-term outcome and recurrence rates in ESUS patients are not very clearly understood. As this clinical construct is a recent definition in the stroke field, the interaction of patients fulfilling inclusion criteria for this condition is a challenge in terms of defining the best therapeutic approach. Some trials have been proposed to study if the recurrence rate in ESUS patients, could be decreased using novel oral anticoagulants,[15–17] with the hypothesis that the current strategy of treatment with antiplatelet may be suboptimal.[15,18,19]

Our study includes an ESUS population with a median age of 44 years, which seems to be quite different from other registries.[12,18] This condition seems to be a distinctive consideration for ethiopathogenic sources in young stroke patients fulfilling ESUS criteria; in a small registry of young ESUS patients, minor embolic sources do not seem to be more prevalent in this population than in strokes of determined cause, which leads to the assumption that further explanations should be identified;[20] despite this, our recurrence rate was not statistically different from the recurrence rate of CS; which leads us to the rationale that even though this patients have been treated with oral antiplatelet drugs, according to current secondary prevention guidelines,[21,22] further therapies and diagnostic procedures should be encouraged among stroke professionals when studying and treating this population.

Risk factors have been widely analyzed among many stroke registries during the last years; ESUS patients could share some typical risk factors, but in ESUS sample, the only risk factor more prevalent was smoking for this group, and other cardiovascular factors (hypertension, diabetes, hypercholesterolemia), were more prevalent in the CS, as expected; therefore the routinary use of therapeutic approaches should be individualized according to the patient needs.

Functional outcome was better in ESUS patients compared to CS, and this finding was seen from discharge until the end of the follow-up; CS was the strongest predictor for poor outcome (HR 3.10; CI 95% 1.96–4.68). Death was significantly higher in CS patients, and none of the ESUS cases presented a fatal outcome. A possible explanation could be related not only to the fact that index infarctions in CS were more severe (according to initial NIHSS and mRS), but also that recurrence in this group was also worst. CS is the leading cause for large infarctions with worst clinical outcome; although embolic cardiac sources for ESUS patients have been proposed, a different emboli production mechanism could be proposed; on the other hand, as our ESUS population is younger than published in other registries, rehabilitation response, neuroplasticity and physical adaptation to disability could be better in this group.[23]

The prognosis of stroke recurrence differs depending of the subtype of stroke. Patients with small-vessel disease have increase risk or death and dementia in the mild and long-term and cognitive impairment is a frequent finding in patients with multiple lacunar infarction recurrences, [24] while in patients with CS stroke, early recurrent embolization is the most important predictor for in-hospital mortality.[25]. In our study, despite recurrence rate in both groups behave similar, two aspects should be analyzed: first, oral anticoagulation for long-term follow-up was not a systematic therapeutic approach for all the CS patients; some of them were under antiplatelet therapy, which is a predisposing factor for recurrence; also INR monitoring could not be obtained systematically from all patients under anticoagulation, as they have medical control in other health centers, to analyze the optimal therapeutic goals; this could explain the reason of oral anticoagulation presents with a HR 8.01 (CI 95% 0.68-94-3) for recurrence, and antiplatelet therapy had the highest risk for recurrence in both groups (HR 24.3; CI 95% 1.82–324.6). The INNN is a referral center for stroke in México City, but patients present different socioeconomic and demographic conditions, and access to medical services for oral anticoagulation monitoring and prescription sometimes is limited. Second, even though ESUS patients from our database are younger, recurrence rate (5.4%) is higher than cryptogenic young stroke registries previously published,[3–6] with the consideration that this is a selective group of patients fulfilling clinical criteria for highly suspicious source of embolism; therefore the rationale of clinical trials assessing the utility of oral anticoagulation vs. current medical therapy (antiplatelet drugs) in this population seems feasible and necessary: NAVIGATE ESUS,[16] RE-SPECT ESUS,[15] and ATTICUS,[17] trials are recruiting patients to analyze this intervention.

Some limitations should be acknowledged in our work; first, this is a retrospective study from our database, with all the drawbacks associated from an observational study analysis, including that we could not made comparisons between clinical expression of the groups due to the lack of detailed clinical information. Second, even though CS is a clinical condition where oral anticoagulation is widely recommended,[21,22] only 64.3% of these patients were treated with this long-term therapy, which could be explained to accessibility to medical services in our country, which is a very important factor to prevent stroke recurrence, and therefore improve long-term good outcome. Also, in our model we used a prospectively collected cohort, and the decision of treatment and long-term follow-up was made from a retrospective fashion.

In conclusion, ESUS patients, while substantially younger, have a similar stroke recurrence rate compared with CS patients, with a lower mortality rate, and better functional outcome on long-term follow-up. These observations support the rationale that meticulous diagnostic work-up for uncommon embolic sources and prolonged cardiac monitoring, as different therapeutic approaches should be established in this population.
